# Women’s decision-making power and undernutrition in their children under age five in the Democratic Republic of the Congo: A cross-sectional study

**DOI:** 10.1371/journal.pone.0226041

**Published:** 2019-12-06

**Authors:** Caroline G. McKenna, Susan A. Bartels, Lesley A. Pablo, Melanie Walker

**Affiliations:** 1 Department of Public Health Sciences, Queen’s University, Kingston, Ontario, Canada; 2 Department of Emergency Medicine, Queen’s University, Kingston, Ontario, Canada; University of Botswana, BOTSWANA

## Abstract

Undernutrition in children remains a major global health issue and the prevalence of undernutrition in children under age five in the Democratic Republic of the Congo (DRC) is among the highest in the world. Both biological and socioeconomic factors contribute to undernutrition, and the literature reports an association between women’s empowerment and lower rates of child undernutrition in sub-Saharan Africa. However, the relationship between women’s decision-making power and child undernutrition is less understood. The objective of this study was to evaluate the association between women’s decision-making power and stunting/wasting in their children under age five in the DRC. This study used cross-sectional data from the 2013–2014 DRC Demographic and Health Survey, from which a sample of 3,721 woman-child pairs were identified. Women were classified as having decision-making power in five decision-making dimensions if they participated in the decision either alone or jointly with their husband or partner or someone else. Child height-for-age and weight-for-height Z-scores were used to determine stunting and wasting, respectively, according to the World Health Organization Child Growth Standards. Multivariate regression analyses demonstrated that none of the five dimensions of decision-making power were associated with stunting or wasting in children. Further research that evaluates women’s decision-making power with more detailed, relevant and context-specific measures is warranted to more accurately investigate women’s decision-making power and undernutrition in children.

## Introduction

### Undernutrition

Undernutrition remains a major global health issue, accounting for 45% of deaths in children under age five who are at the highest risk of becoming undernourished [1]. While malnutrition is often used to describe undernutrition, the term refers to both undernutrition and overweight /obesity [2] and, therefore, undernutrition will be used for the purposes of this research. One third of global child undernutrition occurs in sub-Saharan Africa [3]. The prevalence of undernutrition in children under age five in the Democratic Republic of the Congo (DRC) is among the highest in the world [4], and is estimated to contribute to about 50% of the nation’s under-five mortality rate [1, 5]. Stunting, an indicator of chronic undernutrition, refers to a child who is too short for their age and is associated with prolonged food insecurity or persistent illness. Wasting, however, is an indicator of acute undernutrition, and refers to a child who is too thin for their height [2] and reflects acute food shortages or illnesses. Undernourished children have an increased risk of contracting infectious diseases due to compromised immunity [6], which in turn exacerbates their undernutrition due to decreased appetite or inability to effectively absorb nutrients [7]. In 2014, 43% of children under age five in the DRC suffered from stunting and 8% of children suffered from wasting [8]. The World Health Organization (WHO) reports that stunting prevalence over 30% is considered severe and wasting prevalence over 5% indicates food insecurity [2]. Children who are stunted may suffer severe irreversible physical and cognitive damage [2, 9], and children who are wasted have an increased risk of death if they do not receive timely treatment [2].

UNICEF has outlined the complex and interdependent causes of child undernutrition at the individual level including food intake and disease, at the household level including access to water and proper sanitation, and at the societal level including access to resources [6]. The literature also highlights how both biological and socioeconomic factors contribute to child undernutrition. Socioeconomic factors important in determining nutritional status for children include the societal gender norms, parental occupations, the mother’s level of education, the household’s socioeconomic status, the number of people in the household, the household’s location, and whether the family lives in an urban or rural region [10–13]. Furthermore, a study investigating geographic location and undernutrition in children in the DRC showed that rural/urban location was a predictor of stunting, and demonstrated disparities in undernutrition rates in the eastern provinces, which have been affected by ongoing armed conflict and insecurity [14].

### Women’s decision-making power

Research on the differential preferences regarding resource allocation, including food resources, between men and women suggests that in some low resource settings women are more likely than men to channel resources towards the welfare of their children [15–18]. This allocation of resources, which influences nutritional outcomes, may depend on men’s and women’s ability to negotiate [19], which contributes to women’s decision-making power. In a study in North Kivu, DRC, over half the participants reported that their father had the final say in the majority of household decisions, demonstrating the influence of intergenerational gender inequality in the population [20]. Moreover, gender inequality remains high in the DRC, demonstrated by its low ranking of 36^th^ out of 52 countries on The African Gender Equality Index, which is based on equality in economic opportunities, access to education and reproductive health services, and law and institutions [21]. In 2014, 26% of women in the DRC reported that they did not participate in any decisions regarding major household purchases, visits to their family or relatives, or their own healthcare [8].

Women’s decision-making power has been shown to be associated with child nutritional status in multiple low-income countries, where women with lower decision-making power had higher odds of having children who were undernourished [22–24]. Several studies in sub-Saharan Africa examined women’s empowerment in relation to undernutrition [24–26], with empowerment often being a composite of economic, socio-cultural, legal and/or political variables measured by employment, property ownership, attitudes towards domestic violence, and decision-making power [27]. Comparatively, fewer studies focus specifically on women’s decision-making power and, to our knowledge, no existing study has examined women’s decision-making power and child undernutrition in the DRC. Furthermore, although the DHS uses standardized measures of decision-making across many countries, there is no uniform definition of decision-making power in the literature and it is unclear which dimensions of decision-making, if any, predict child undernutrition.

The DHS measures five dimensions of women’s decision-making: decisions regarding how to spend her own income, how to spend her husband’s income, her own healthcare, major household purchases, and visits to family and relatives. Literature suggests that these five decision-making indicators may be relevant for child undernutrition. For instance, women’s access to and control of financial resources (i.e. decision-making regarding her own and/or husband’s income spending) has been associated with improvements in child nutrition [11]. Furthermore, women’s participation in health-care decision-making may be a proxy measure for a household’s overall access to healthcare (a question not directly asked in the DHS women’s questionnaire) which has shown to be protective against stunting prevalence in Tanzania [28]. In addition, a woman’s decision-making regarding visits to her family and relatives demonstrates her mobility autonomy, which allows for greater independence and may promote access to broader resources for her children. For instance, a study from India showed that mothers who did not need permission to go to the market were less likely to have a stunted child [23].

The objectives of this study are, therefore, to: 1) evaluate the association between five dimensions of women’s decision-making power and undernutrition in their children under age five in the DRC, and 2) to determine which, if any, of these five decision-making dimensions are the strongest predictors of child undernutrition.

## Methods

### Data source and study population

The 2013–2014 DRC Demographic and Health Survey (DHS) is a nationally representative cross-sectional survey that includes a Women’s Questionnaire and a Biomarker Survey. Data was collected between August 2013 and February 2014 by trained local interviewers led by survey supervisors. DHS sampling is designed to be representative at the national and provincial level, as well as rural and urban regional levels [8]. Multi-stage stratified cluster sampling was used to identify a stratified sample of enumeration areas, from which 536 clusters were selected and 18,190 households were surveyed. Detailed methodology for the 2013–2014 DRC DHS can be freely accessed from the DHS Program website [29].

**Fig 1:**
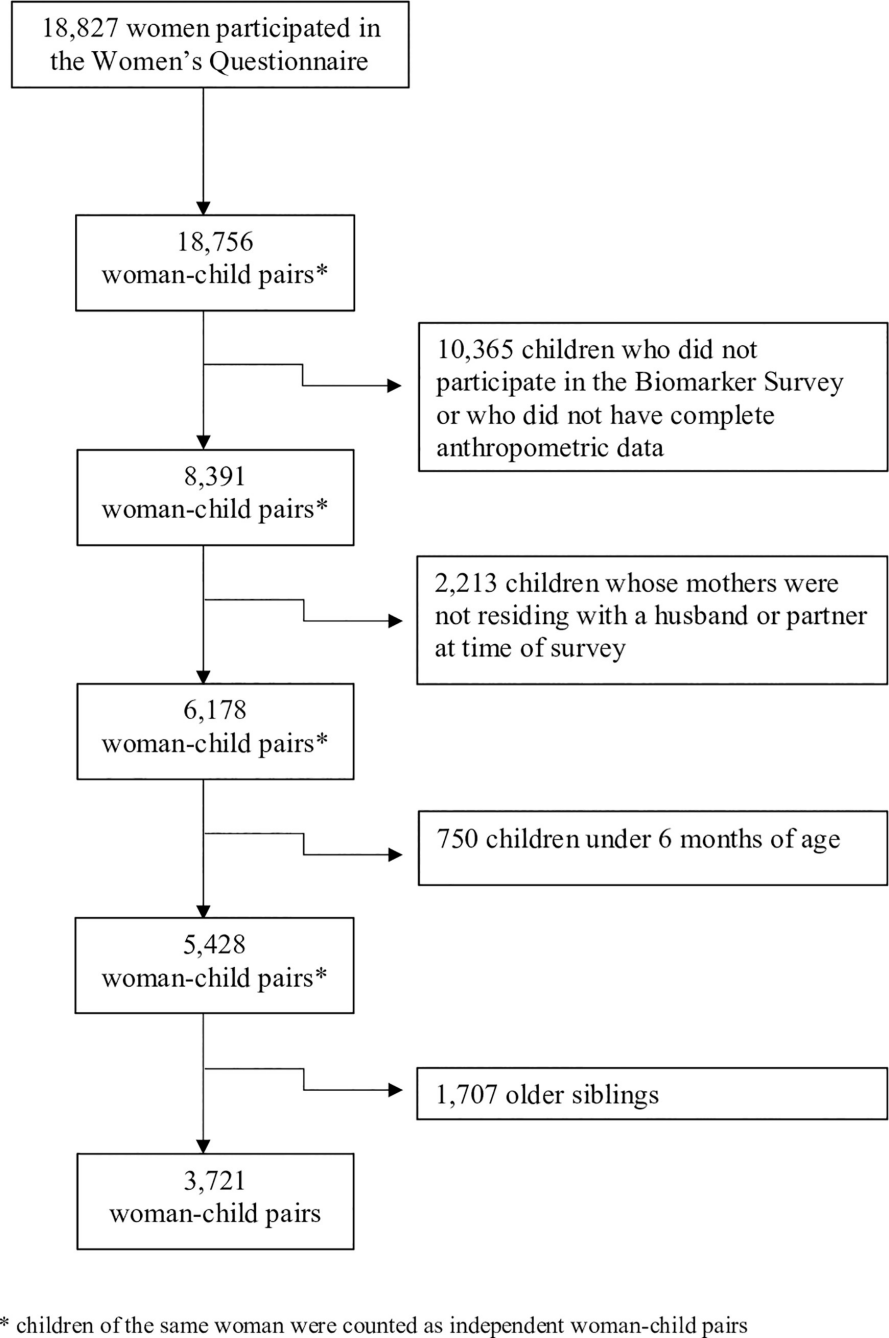
Flowchart of sample selection. A final sample of 3,721 woman-child pairs was selected based on the following inclusion criteria: 1) women age 15–49 and their children age 6–59 months, 2) children with complete anthropometric data, 3) women living with a husband or partner at time of survey, 4) selection of youngest child per woman.

A total of 18,827 women between the ages of 15 and 49 participated in the Women’s Questionnaire with a 99% response rate. In a sub-sample of every other household, women were eligible to complete the Biomarker Survey, where anthropometric measurements of their children under age five were taken. The children’s anthropometric data were linked to their mother’s survey responses by unique identifiers. The following inclusion criteria were used to select the final sample: women who were living with a husband or with a partner as if they were married at the time of the survey, and their youngest child aged 6–59 months had complete anthropometric data. Women who did not have any children under age five were excluded. To avoid cases where women were the de facto primary decision-maker, women who were living alone were also excluded. Children under the age of 6 months were excluded due to the low risk of stunting in this age group [30]. Finally, the youngest child of each eligible woman was selected, consistent with other studies involving woman-child pairs [25, 31]. Fig 1 illustrates the selection of study participants which resulted in a final sample of 3,721 woman-child pairs.

### Measures

The exposure of interest, women’s decision-making power, was determined by the participants’ responses to five questions, which asked women who usually makes decisions regarding: 1) the respondent’s own income spending, 2) her husband / partner’s income spending, 3) the respondent’s own healthcare, 4) major household purchases, and 5) visits to family and relatives (S1 Table). A woman was considered to participate in decision-making if she indicated that she usually makes the decision alone, jointly with her husband or partner, or jointly with someone else. Using DHS data, it was not possible to measure the nature of the power balance within joint decisions, for example whether the decision was driven primarily by the woman or her husband / partner. The number of women who made decisions alone was also quite low in the overall study population. For example, for decisions regarding the respondent’s own healthcare, only 261 women participated in the decision alone, 1405 women participated in the decision with her husband or partner, and 2053 women did not participate in the decision. In order to maximize statistical power, we dichotomized our exposure variable as women who participated (solely or jointly) or did not participate in decision making on the five independent variables of interest.

The outcome of interest, stunting and wasting in their children under age five, was determined from the height-for-age (HAZ) Z-scores and weight-for-height (WHZ) Z-scores respectively, as calculated from the children’s age in years, weight in kilograms and height in centimeters. Children were considered stunted if their HAZ Z-score was more than 2 standard deviations (SDs) below the median of the WHO Child Growth Standards reference population. Children were considered wasted if their WHZ Z-score was more than 2 SDs below the median of the WHO Child Growth Standards reference population [32]. Since stunting and wasting are primarily indicative of chronic and acute malnutrition, respectively, they were the measures of interest in this study. Underweight was not included as it is indicative of both chronic and acute malnutrition together, and may not clearly delineate potential causal relationships if an association was observed.

Covariates of interest included child’s sex, child’s age, mother’s education, mother’s age, preceding birth interval, number of children under age five in the household, number of household members, province, place of residence, and household socioeconomic status (S1 Table).

### Statistical analysis

Differences in stunting and wasting prevalence across demographic characteristics of interest were evaluated using chi-square tests. Logistic regression was used to evaluate the association between women’s decision-making power and stunting/wasting in their children under age five. Bivariate logistic regression was used to assess the unadjusted association between each of the five individual dimensions of decision-making with both stunting and wasting outcomes. Covariates associated with stunting / wasting at *p* < 0.10 from the chi-square tests were included in the initial multivariate logistic regression model. The final multivariate regression models were created controlling for 1) known risk factors of child undernutrition identified in the literature (child’s age, child’s sex, and household socioeconomic status) and 2) confounding variables identified per model using a backwards selection process at *p* < 0.10 for each of the five decision-making dimensions under study (province, place of residence, mother’s age, mother’s education, and/or number of children under 5 in the household). Finally, a stratified analysis was conducted to investigate the association between women’s decision-making and child stunting / wasting in the eastern and western provinces of the DRC, where conflict-affected eastern provinces included North and South Kivu, Maniema, Katanga, and Orientale [33] and western provinces were comprised of the remaining.

IBM SPSS Statistics (version 25.0) was used to conduct data analysis and results were considered statistically significant if *p* < 0.05. To account for the impact of the underlying complex sampling design on logistic regression parameters, data were weighted using the SPSS Complex Samples Package [34]. Sample weights provided by DHS were used to adjust for differences in sampling probabilities.

### Ethical considerations

The DHS Program specifies that informed consent was obtained from study participants before the interviews were conducted, and all data were de-identified at source before being shared with our research team. This study protocol was approved by the Queen’s University Health Sciences Research Ethics Board (HSREB) (protocol #6025377).

## Results

### Descriptive statistics

A total of 3,721 woman-child pairs were included in the study. In this sample, 35.2% and 9.2% of children were stunted and wasted, respectively, according to WHO Child Growth Standards.

Table 1 outlines the descriptive statistics of the study sample disaggregated by stunting and wasting status of the child. To summarize, the stunted group was observed to have a higher proportion of male children (*p* < 0.01), children greater than one year of age (*p* < 0.001), children whose mothers were without higher education (*p* < 0.001), children whose mothers were between 35 and 49 years old (*p* = 0.02), and children with a preceding birth interval of < 24 months (*p* < 0.01). Additionally, the stunted and non-stunted groups differed in the number of children under five in the household (*p* = 0.01), the province (*p* < 0.001), and place of residence (*p* < 0.001). The stunted group had a higher proportion of children in the two poorest quintiles compared to the non-stunted group (*p* < 0.001). The wasted group had a higher proportion of male children (*p* < 0.01), children under one year of age (*p* = 0.04), and children whose mothers had only a primary school education (*p* = 0.04). Province (*p* = 0.03), place of residence (*p* = 0.01), and wealth index quintile (*p* < 0.01) also differed between the wasted and non-wasted groups.

**Table 1:** Demographic characteristics of the study population (woman-child pairs) by nutritional status.

	Overall		Stunted n = 1310		Non-Stunted n = 2411		*p*-value	Wasted n = 344		Non-Wasted n = 3377		*p*-value
	n	%	n	%	n	%		n	%	n	%	
**Own Income**							0.71					0.37
Participates	1616	64.2	577	64.3	1039	64.1		130	61.0	1486	64.4	
Does not participate	903	35.8	320	35.7	583	35.9		83	39.0	820	35.6	
**Husband’s Income**							0.07					0.12
Participates	2043	55.5	694	53.4	1349	56.6		172	50.4	1871	56.0	
Does not participate	1640	44.5	606	46.6	1034	43.4		169	49.6	1471	44.0	
**Own Healthcare**							0.84					0.56
Participates	1666	44.8	593	45.3	1073	44.5		143	41.7	1523	45.1	
Does not participate	2053	55.2	716	54.7	1337	55.5		200	58.3	1853	54.9	
**Household Purchases**							0.54					0.97
Participates	2140	57.6	744	56.8	1396	58.0		195	56.7	1945	57.7	
Does not participate	1576	42.4	565	43.2	1011	42.0		149	43.3	1427	42.3	
**Visits to Family**							0.96					0.86
Participates	1865	50.2	658	50.3	1207	50.1		168	48.8	1697	50.3	
Does not participate	1853	49.8	650	49.7	1203	49.9		176	51.2	1677	49.7	
**Child’s Sex**							<0.01*					<0.01*
Male	1859	50.0	728	55.6	1131	46.9		200	58.1	1659	49.1	
Female	1862	50.0	582	44.4	1280	53.1		144	41.9	1718	50.9	
**Child’s Age (years)**							<0.001*					0.04*
0	1356	36.4	242	18.5	1114	46.2		148	43.0	1208	35.8	
1	1173	31.5	451	34.4	722	29.9		101	29.4	1072	31.7	
2	727	19.5	384	29.3	343	14.2		63	18.3	664	19.7	
3	308	8.3	165	12.6	143	5.9		25	7.3	283	8.4	
4	157	4.2	68	5.2	89	3.7		7	2.0	150	4.4	
**Mother’s education**							<0.001*					0.04*
None	840	22.6	349	26.6	491	20.4		70	20.3	770	22.8	
Primary	1663	44.7	620	47.3	1043	43.3		178	51.7	1485	44.0	
Secondary	1177	31.6	336	25.6	841	34.9		94	27.3	1083	32.1	
Higher	41	1.1	5	0.4	36	1.5		2	0.6	39	1.2	
**Mother’s age (years)**							0.02*					0.70
15–19	217	5.8	64	4.9	153	6.3		23	6.7	194	5.7	
20–24	753	20.2	261	19.9	492	20.4		61	17.7	692	20.5	
25–29	1046	28.1	350	26.7	696	28.9		105	30.5	941	27.9	
30–34	720	19.3	252	19.2	468	19.4		62	18.0	658	19.5	
35–39	579	15.6	211	16.1	368	15.3		53	15.4	526	15.6	
40–44	317	8.5	137	10.5	180	7.5		29	8.4	288	8.5	
45–49	89	2.4	35	2.7	54	2.2		11	3.2	78	2.3	
**Preceding Birth Interval (months)**			n = 1148		n = 2075		<0.01*	n = 291		n = 2932		0.07
0–23	737	19.8	301	23.0	436	18.1		79	23.0	658	19.5	
≥ 24	2846	66.8	847	64.7	1639	68.0		212	61.6	2274	67.3	
Missing	498	13.4	162	12.4	336	13.9		53	15.4	445	13.2	
**Number of children under 5 in household**							0.01*					0.15
≤1	1413	38.0	532	40.6	881	36.5		114	33.1	1299	38.5	
2	1718	46.2	598	45.6	1120	46.5		170	49.4	1548	45.8	
≥3	590	15.9	180	13.7	410	17.0		60	17.4	530	15.7	
**Number of people in household**							0.63					0.95
≤5	1418	38.1	490	37.4	928	38.5		133	38.7	1285	38.1	
6–10	2030	54.6	728	55.6	1302	54.0		185	53.8	1845	54.6	
>10	273	7.3	92	7.0	181	7.5		26	7.6	247	7.3	
**Province**							<0.001*					0.03*
Kinshasa	195	5.2	27	2.1	168	7.0		9	2.6	186	5.5	
Bandundu	528	14.2	176	13.4	352	14.6		59	17.2	469	13.9	
Bas-Congo	177	4.8	69	5.3	108	4.5		16	4.7	161	4.8	
Equateur	581	15.6	185	14.1	396	16.4		54	15.7	527	15.6	
Kasi-Occidental	321	8.6	129	9.8	192	8.0		26	7.6	295	8.7	
Kasi-Oriental	431	11.6	168	12.8	263	10.9		44	12.8	387	11.5	
Katanga	473	12.7	189	14.4	284	11.8		47	13.7	426	12.6	
Maniema	221	5.9	73	5.6	148	6.1		31	9.0	190	5.6	
North-Kivu	214	5.8	87	6.6	127	5.3		14	4.1	200	5.9	
Orientale	358	9.6	113	8.6	245	10.2		32	9.3	326	9.7	
South-Kivu	222	6.0	94	7.2	128	5.3		12	3.5	210	6.2	
**Place of residence**							<0.001*					0.01*
Urban	1021	27.4	265	20.2	756	31.4		73	21.2	948	28.1	
Rural	2700	72.6	1045	79.8	1655	68.6		271	78.8	2429	71.9	
**Household socioeconomic status**							<0.001*					<0.01*
Poorest	949	25.5	366	27.9	583	24.2		95	27.6	854	25.3	
Poorer	918	24.7	372	28.4	546	22.6		89	25.9	829	24.5	
Middle	798	21.4	290	22.1	508	21.1		87	25.3	711	21.1	
Richer	601	16.2	197	15.0	404	16.8		52	15.1	549	16.3	
Richest	455	12.2	85	6.5	370	15.3		21	6.1	434	12.9	

* *p*-values are based on chi-square tests and statistically significant if *p* < 0.05

Data are unweighted. Percentage sum discrepancies are due to rounding.

Women’s participation, solely or jointly, in the five decision-making variables ranged from a low of 41.7% for decisions regarding her own healthcare to a high of 64.4% for decisions regarding her own income. Neither the stunted and non-stunted groups, nor the wasted and non-wasted groups, differed in terms of the decision-making power of their mothers (*p* > 0.05).

**Fig 2:**
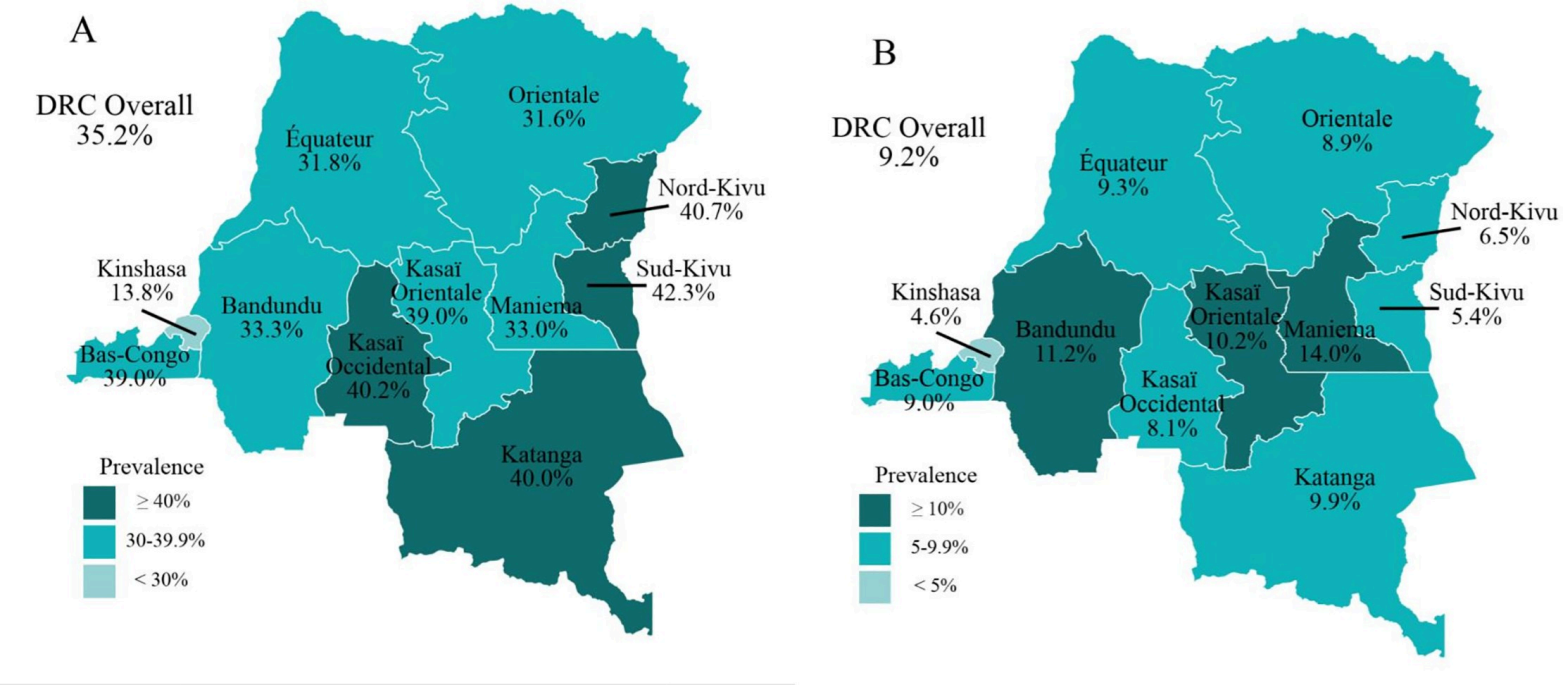
Map of the Democratic Republic of the Congo depicting the prevalence rates of stunting and wasting in children under age five by province. **A)** shows the percent of children under age five who are stunted by province. **B)** shows the percent of children under age five who are wasted by province.

Fig 2 shows the prevalence of stunting and wasting in children under age five by province. Of note, eastern provinces including Katanga, North-Kivu and South-Kivu had the highest rates of stunting, while the national capital, Kinshasa, had the lowest rates of both stunting and wasting.

### Logistic regression

Table 2 shows the weighted unadjusted odds ratios of the bivariate logistic regression of the covariates and stunting / wasting. At the bivariate level, women’s decision-making was not associated with stunting / wasting. Tables 3 and 4 show the results of the multivariate logistic regression for each of the five decision making variables on the outcomes of stunting and wasting, respectively, while controlling for confounding variables. There were no observed associations between women’s decision-making power and stunting or wasting in their children under age five after controlling for confounding variables.

**Table 2:** Weighted unadjusted odd ratios for covariates and stunting / wasting.

	Stunting	Wasting
**Decision Making**		
**Own income**		
Participates	1.0	1.0
Does not participate	1.06 (0.78, 1.43)	1.29 (0.74, 2.23)
**Husband’s income**		
Participates	1.0	1.0
Does not participate	1.19 (0.98, 1.44)	1.33 (0.93, 1.92)
**Own healthcare**		
Participates	1.0	1.0
Does not participate	1.02 (0.84, 1.23)	1.11 (0.78, 1.58)
**Major household purchases**		
Participates	1.0	1.0
Does not participate	1.07 (0.87, 1.31)	0.99 (0.74, 1.35)
**Visits to family**		
Participates	1.0	1.0
Does not participate	1.01 (0.83, 1.22)	0.97 (0.72, 1.31)
**Child’s Sex**		
Male	1.0	1.0
Female	0.78 (0.66, 0.93)*	0.79 (0.57, 1.09)
**Child’s Age (years)**		
0	1.0	1.0
1	3.19 (2.40, 4.24)*	0.61 (0.41, 0.90)*
2	5.32 (3.74, 7.57)*	0.71 (0.44, 1.15)
3	5.77 (4.01, 8.31)*	0.72 (0.40, 1.31)
4	4.36 (2.69, 7.04)*	0.25 (0.09, 0.73)*
**Mother’s education**		
None	1.0	1.0
Primary	0.93 (0.74, 1.16)	1.49 (1.04, 2.13)*
Secondary	0.57 (0.45, 0.73)*	0.87 (0.54, 1.40)
Higher	0.13 (0.04, 0.45)*	0.41 (0.08, 2.17)
**Mother’s age (years)**		
15–19	1.0	1.0
20–24	1.63 (1.05, 2.53)*	0.83 (0.39, 1.77)
25–29	1.33 (0.87, 2.04)	0.96 (0.47, 1.97)
30–34	1.46 (0.95, 2.23)	1.06 (0.49, 2.28)
35–39	1.59 (1.01, 2.51)*	1.46 (0.66, 3.27)
40–44	1.79 (1.07, 2.97)*	1.57 (0.65, 3.81)
45–49	1.59 (0.78, 3.26)	1.07 (0.36, 3.20)
**Preceding Birth Interval**		
0–23	1.0	1.0
≥ 24	0.79 (0.62, 1.00)	0.92 (0.62, 1.37)
**Number of children under 5 in household**		
≤1	1.0	1.0
2	0.89 (0.70, 1.13)	1.26 (0.86, 1.84)
≥3	0.77 (0.57, 1.03)	1.09 (0.71, 1.67)
**Household size**		
≤5	1.0	1.0
6–10	1.19 (0.98, 1.44)	1.01 (0.68, 1.49)
>10	0.96 (0.64, 1.44)	0.90 (0.53, 1.55)
**Province**		
Kinshasa	1.0	1.0
Bandundu	2.73 (1.57, 4.73)*	3.01 (1.32, 6.83)*
Bas-Congo	4.40 (2.09, 9.26)*	2.31 (0.83, 6.48)
Equateur	2.79 (1.63, 4.78)*	1.58 (0.68, 3.64)
Kasi-Occidental	4.29 (2.33, 7.88)*	1.99 (0.77, 5.12)
Kasi-Oriental	4.03 (2.36, 6.89)*	1.75 (0.74, 4.14)
Katanga	3.50 (1.98, 6.19)*	1.83 (0.83, 4.07)
Maniema	2.77 (1.48, 5.18)*	4.41 (1.75, 11.07)*
North-Kivu	3.44 (1.87, 6.33)*	1.16 (0.34, 3.93)
Orientale	3.08 (1.71, 5.57)*	1.41 (0.56, 3.55)
South-Kivu	5.09 (2.65, 9.76)*	1.40 (0.58, 3.42)
**Place of Residence**		
Urban	1.0	1.0
Rural	2.07 (1.64, 2.60)*	1.64 (1.13, 2.38)*
**Household socioeconomic status**		
Poorest	1.0	1.0
Poorer	1.02 (0.77, 1.35)	0.91 (0.57, 1.43)
Middle	0.97 (0.74, 1.28)	1.03 (0.68, 1.58)
Richer	0.73 (0.51, 1.05)	0.87 (0.54, 1.40)
Richest	0.32 (0.22, 0.46)*	0.41 (0.23, 0.73)*

* statistically significant based on 95% confidence interval not crossing 1.0

**Table 3:** Adjusted odds ratios for the outcome of stunting.

Covariate	Regarding her own income^a^	Regarding her husband’s income^b^	Regarding her own health care^b^	Regarding major household purchases^b^	Regarding visits to family^b^
**Participates in decision making**					
Yes	1.0	1.0	1.0	1.0	1.0
No	0.89 (0.63, 1.26)	1.14 (0.94, 1.39)	1.10 (0.89, 1.35)	1.08 (0.88, 1.33)	1.01 (0.82, 1.24)
**Child’s sex**					
Male	1.0	1.0	1.0	1.0	1.0
Female	0.68 (0.53, 0.86)*	0.79 (0.65, 0.96)*	0.79 (0.65, 0.96)*	0.79 (0.65, 0.95)*	0.78 (0.65, 0.95)*
**Child’s age in years**					
0	1.0	1.0	1.0	1.0	1.0
1	2.94 (2.16, 4.00)*	3.42 (2.60, 4.51)*	3.37 (2.56, 4.45)*	3.39 (2.56, 4.48)*	3.36 (2.54, 4.44)*
2	5.54 (3.81, 8.06)*	5.86 (4.16, 8.26)*	5.76 (4.07, 8.14)*	5.81 (4.10, 8.23)*	5.73 (4.05, 8.10)*
3	7.71 (4.49, 13.24)*	6.77 (4.58, 10.01)*	6.81 (4.64, 10.12)*	6.83 (4.64, 10.06)*	6.76 (4.59, 9.97)*
4	7.73 (4.21, 14.21)*	5.50 (3.38, 8.94)*	5.37 (3.32, 8.67)*	5.35 (3.31, 8.65)*	5.37 (3.32, 8.69)*
**Mother’s age in years**					
15–19	1.0				
20–24	0.92 (0.54, 1.56)				
25–29	0.65 (0.39, 1.07)				
30–34	0.74 (0.44, 1.23)				
35–39	0.69 (0.38, 1.27)				
40–44	0.64 (0.34, 1.18)				
45–49	0.23 (0.09, 1.57)*				
**Number of children under 5 in household**					
≤1	1.0				
2	1.38 (0.93, 2.06)				
≥3	1.80 (1.20, 2.70)*				
**Province**					
Kinshasa	1.0	1.0	1.0	1.0	1.0
Bandundu	1.26 (0.59, 2.73)	0.99 (0.50, 1.93)	1.01 (0.51, 1.97)	1.01 (0.52, 1.97)	1.01 (0.52, 1.97)
Bas-Congo	2.53 (1.06, 6.04)*	2.20 (1.01, 4.80)*	2.25 (1.03, 4.92)*	2.38 (1.10, 5.13)*	2.26 (1.03, 4.95)*
Equateur	1.30 (0.60, 2.78)	0.93 (0.48, 1.83)	0.94 (0.48, 1.84)	0.95 (0.49, 1.85)	0.95 (0.49, 1.86)
Kasi-Occidental	1.87 (0.82, 4.23)	1.90 (0.91, 3.97)	1.90 (0.91, 3.97)	1.90 (0.91, 3.96)	1.91 (0.92, 4.00)
Kasi-Oriental	2.10 (1.00, 4.41)*	1.82 (0.95, 3.47)	1.84 (0.97, 3.52)	1.85 (0.97, 3.53)	1.87 (0.98, 3.58)
Katanga	2.41 (1.09, 5.34)*	2.04 (1.04, 4.00)*	2.07 (1.06, 4.04)*	2.07 (1.06, 4.06)*	2.08 (1.07, 4.06)*
Maniema	0.70 (0.23, 2.10)	0.97 (0.45, 2.10)	0.98 (0.45, 2.11)	0.98 (0.45, 2.11)	0.99 (0.46, 2.14)
North-Kivu	1.74 (0.71, 4.23)	1.67 (0.79, 3.54)	1.70 (0.80, 3.60)	1.68 (0.80, 3.56)	1.68 (0.79, 3.55)
Orientale	1.47 (0.65, 3.35)	1.20 (0.59, 2.43)	1.22 (0.60, 2.47)	1.23 (0.61, 2.49)	1.21 (0.60, 2.46)
South-Kivu	3.67 (1.62, 8.33)*	2.82 (1.35, 5.90)*	2.86 (1.37, 5.97)*	2.92 (1.41, 6.05)*	2.82 (1.35, 5.88)*
**Household economic status**					
Poorest	1.0	1.0	1.0	1.0	1.0
Poorer	0.91 (0.63, 1.31)*	0.91 (0.68, 1.22)	0.91 (0.68, 1.23)	0.91 (0.68, 1.23)	0.91 (0.68, 1.22)
Middle	0.80 (0.56, 1.14)*	0.84 (0.64, 1.11)	0.83 (0.63, 1.10)	0.84 (0.64, 1.11)	0.83 (0.63, 1.09)
Richer	0.56 (0.36, 0.89)*	0.58 (0.40, 0.84)*	0.57 (0.40, 0.83)*	0.57 (0.40, 0.83)*	0.57 (0.39, 0.83)*
Richest	0.28 (0.17, 0.49)*	0.29 (0.18, 0.46)*	0.28 (0.18, 0.45)*	0.28 (0.18, 0.45)*	0.28 (0.18, 0.44)*

* Represents a statistically significant finding

^a^ controlling for child’s sex, child’s age, household socioeconomic status, province, mother’s age, number of children under five in household

^b^ controlling for child’s sex, child’s age, household socioeconomic status, and province

**Table 4:** Adjusted odds ratios for the outcome of wasting.

Covariate	Regarding her own income^a^	Regarding her husband’s income^b^	Regarding her own health care^b^	Regarding major household purchases^b^	Regarding visits to family^b^
**Participates in decision making**					
Yes	1.0	1.0	1.0	1.0	1.0
No	1.10 (0.68, 1.79)	1.21 (0.85, 1.74)	1.07 (0.75, 1.53)	0.94 (0.69, 1.28)	0.93 (0.69, 1.24)
**Child’s sex**					
Male	1.0	1.0	1.0	1.0	1.0
Female	0.88 (0.59, 1.30)	0.77 (0.56, 1.07)	0.77 (0.56, 1.06)	0.77 (0.56, 1.06)	0.77 (0.56, 1.06)
**Child’s age in years**					
0	1.0	1.0	1.0	1.0	1.0
1	0.55 (0.32, 0.93)*	0.58 (0.38, 0.87)*	0.59 (0.39, 0.88)*	0.59 (0.39, 0.88)*	0.58 (0.39, 0.88)*
2	0.63 (0.35, 1.12)	0.68 (0.42, 1.10)	0.68 (0.42, 1.09)	0.67 (0.41, 1.09)	0.67 (0.42, 1.09)
3	0.47 (0.20, 1.11)	0.71 (0.39, 1.29)	0.71 (0.39, 1.29)	0.70 (0.38, 1.29)	0.70 (0.38, 1.29)
4	0.31 (0.10, 0.92)*	0.26 (0.09, 0.75)*	0.26 (0.09, 0.74)*	0.26 (0.09, 0.74)	0.26 (0.09, 0.74)*
**Mother’s education**					
None	1.0	1.0	1.0	1.0	1.0
Primary	1.62 (1.03, 2.55)*	1.55 (1.09, 2.20)*	1.54 (1.08, 2.18)*	1.53 (1.08, 2.16)*	1.54 (1.09, 2.18)*
Secondary	0.91 (0.43, 1.92)	1.07 (0.66, 1.74)	1.05 (0.64, 1.74)	1.04 (0.64, 1.70)	1.06 (0.64, 1.73)
High	1.63 (0.24, 11.25)	0.77 (0.14, 4.29)	0.78 (0.12, 4.30)	0.75 (0.14, 4.20)	0.75 (0.14, 4.18)
**Province**					
Kinshasa	1.0				
Bandundu	1.20 (0.23, 6.12)				
Bas-Congo	1.04 (0.19, 5.72)				
Equateur	0.44 (0.08, 2.24)				
Kasi-Occidental	0.70 (0.13, 3.60)				
Kasi-Oriental	0.52 (0.11, 2.61)				
Katanga	0.81 (0.16, 4.18)				
Maniema	2.50 (0.45, 13.74)				
North-Kivu	0.36 (0.06, 2.36)				
Orientale	0.48 (0.08, 2.76)				
South-Kivu	0.47 (0.09, 2.41)				
**Household economic status**					
Poorest	1.0	1.0	1.0	1.0	1.0
Poorer	1.26 (0.79, 2.01)	0.92 (0.56, 1.49)	0.92 (0.57, 1.49)	0.91 (0.57, 1.47)	0.91 (0.57, 1.47)
Middle	1.32 (0.79, 2.21)	1.01 (0.66, 1.56)	1.04 (0.68, 1.58)	1.04 (0.68, 1.58)	1.03 (0.68, 1.57)
Richer	1.40 (0.74, 2.63)	0.89 (0.53, 1.48)	0.89 (0.54, 1.46)	0.87 (0.53, 1.43)	0.87 (0.53, 1.43)
Richest	0.39 (0.12, 1.34)	0.48 (0.25, 0.91)*	0.46 (0.24, 0.86)*	0.45 (0.24, 0.85)*	0.45 (0.24, 0.85)*

* Represents a statistically significant finding

^a^ controlling for child’s sex, child’s age, household socioeconomic status, mother’s education, and province

^b^ controlling for child’s sex, child’s age, household socioeconomic status, and mother’s education

A stratified analysis investigating the association between women’s decision-making power and stunting / wasting in their children in the eastern and western provinces of the DRC was conducted. In western provinces, women who did not participate in decisions regarding her husband’s income had higher odds of having a child who was stunted than women who participated in this decision (OR = 1.28, 95% CI: (1.00, 1.63)) (S2 Table).

A post-hoc analysis to assess the robustness of results with a different exposure classification was also conducted. Specifically, we examined: 1) the association between joint decision-making and women’s decision-making alone compared with on the outcomes of interest, and 2) the association between no participation in decision making and women’s decision making alone compared with on the outcomes of interest. Women who made decisions regarding her husband’s income jointly with her husband / partner/ someone else had higher odds of having a stunted child than women who made such decisions alone (OR = 1.59, 95% CI: (1.06, 2.40)) (S3 Table). In addition, women who did not participate in decisions regarding her husband’s income at all had higher odds of having a stunted child than women who made such decisions alone (OR = 1.71, 95% CI: (1.14, 2.57)) (S4 Table).

## Discussion

This study used a large, nationally representative sample to investigate the association between women’s decision-making power and stunting / wasting in their children under age five.

In the current sample, 35.2% and 9.2% of children were stunted and wasted, respectively, despite national population rates of 43% and 8% [8]. Our lower prevalence of stunting may result from the fact that we only included women who were living with a husband / partner, and children whose mothers live alone may disproportionately account for greater stunting rates. Inclusion of the youngest child per woman may explain the slightly higher prevalence of wasting in the sample as higher birth order has shown to be a risk factor for wasting [35]. However, it should be noted that wasting is an indicator of acute undernutrition and is more heavily influenced by seasonality, acute food insecurity, and acute diseases than stunting [2], and thus may be a less reliable measure of child undernutrition especially in a cross-sectional study. Eastern DRC provinces including Katanga, North-Kivu and South-Kivu were observed to have the highest rates of stunting, likely due to decades of armed conflict and ongoing insecurity.The stunted and non-stunted groups differed significantly with regards to child’s sex, child’s age, mother’s education, mother’s age, preceding birth interval, number of children under five in the household, province, place of residence, and household socioeconomic status. These findings are consistent with those of Kismul *et al*. who also found that the prevalence of stunting among Congolese children was higher in boys, in children of mothers with little or no education, in children of households with the lowest socioeconomic status, and in children living in rural areas [36].

The wasted and non-wasted groups also differed significantly with regards to child’s sex, child’s age, mother’s education, province, place of residence, and household socioeconomic status, with the finding regarding age being consistent with that of Ross-Suits who noted lower rates of wasting in children above age two [37]. Women’s participation in decision-making regarding her husband / partner’s income was lower in the wasted group than the non-wasted group, which is consistent with a study in Ethiopia where rates of wasting in children were higher in households where mothers were not able to use money [38].

Collectively, the five dimensions of decision-making were intended to measure women’s overall autonomy at the individual, household, and societal level. At the individual level, women’s participation in decision-making demonstrates internal empowerment and self-determination [17]. At the household level, women’s participation in decision-making indicates intra-household authority and greater intra-household gender equality [15]. At the societal level, women’s participation in decision-making indicates social support in her community [21]. For example, a woman who is able to make decisions about her own healthcare means not only that she can participate in decision-making itself, but also that she lives in a community where she can access care independently, including travel to the health facility, ability to use money for this purpose, and being treated by healthcare providers without her husband.

In this study, no association was observed between any of the five decision-making variables and stunting or wasting in children under age five. This was unexpected since other studies in low-resource settings have identified decision-making as a predictor of stunting, wasting, and underweight in children [22–25]. Furthermore, a study in South Kivu, DRC found that lack of women’s household decision-making power negatively influenced feeding practices in children under age two [39], which is a key determinant of under-five nutritional status.

Based on the literature, other measures of decision-making, including decisions regarding women’s mobility [23], daily household purchases [40], and child healthcare [41] may be more relevant markers of decision making in the house in relation to child undernutrition, however, such information was not available from the 2013/14 DHS data. We did observe that some covariates were significant predictors of stunting and wasting. Specifically, child’s sex, child’s age, household socioeconomic status, mother’s age, number of children under five in the household, province, and place of residence were associated with child stunting. In addition, child’s age, household socioeconomic status, mother’s education, and place of residence were associated with child wasting. These variables may be better predictors of child undernutrition than women’s decision-making power, as collected by the DHS.

Our findings are largely consistent with a study that used the 2004–2005 Tanzania-DHS, which also found no association between women’s decision-making regarding household purchases and visits to family and undernutrition in their children [37]. However, decision-making power regarding the respondent’s own healthcare was associated with a lower odds of child stunting. This inconsistency may be due to differential access to health services in the DRC and Tanzania, which may affect the implications of this decision in these two contexts. Our identification of covariates that were associated with stunting are consistent with findings by Kismul *et al*. who also found child’s sex, child’s age, household socioeconomic status, and province to be associated with stunting [36].

The stratified analysis of eastern and western provinces of the DRC showed that in western provinces, women who did not participate in decisions regarding her husband’s income had higher odds of having a child who was stunted than women who participated in this decision. It may be that this association is evident in eastern provinces as well, but that the ongoing armed conflict in these provinces may be dampening the relationship between decision-making power and child stunting. This finding provides grounds for future research to examine child undernutrition separately in eastern versus western provinces.

The lack of association between women’s participation in decisions regarding her own income and stunting / wasting could result from the way the DHS survey question was structured. In the sample of 3721 participants, 852 women (22.9%) said that they were not currently working. When asked who usually makes decisions regarding how to spend her own income, the DHS survey did not provide a “not applicable” response choice. Consequently, the number of women who reported that they do not participate in these decisions may be overestimated because this group likely also includes women for whom this question was not applicable (due to the absence of any income). In comparison, the question that asks women who usually makes decisions regarding how to spend her husband’s income, the DHS provided “husband has no income” as a response option which would avoid this over-estimation.

Furthermore, the DHS’ internationally standardized decision-making questions may have also contributed to our finding that there was no association between women’s decision-making and child undernutrition. After 2007, the DHS reduced the collection of decision making variables, including decisions regarding daily purchases, which may make it difficult to completely understand the role of decision making on child nutrition outcomes. In light of these finding, the DHS may consider including more comprehensive questions regarding women’s decision-making, including who has the final say in joint decision-making, decisions regarding child healthcare, and decisions regarding women’s mobility, to better delineate any potential relationships between decision-making and child undernutrition. In addition, large, multi-country surveys may exclude culturally-sensitive dimensions of decision-making that are relevant to child undernutrition. Thus, context-specific questions accounting for the sociocultural circumstances in the DRC may be required to measure decision-making more accurately. For example, while mother’s decision-making regarding her own healthcare has been shown to be strongly associated with her own nutritional status [41], it was not found to be associated with child undernutrition in this study. Decision-making regarding her child’s healthcare, for instance, may be a better predictor of child undernutrition and may be worth exploring in future research.

It may also be that biological factors are more strongly associated with child undernutrition than women’s participation in decision-making. For instance, child’s age and sex, child’s birth order, preceding birth interval, how long the child was breastfed, and the mother’s age and nutritional status during pregnancy are all important determinants of a child’s nutritional status [35, 42–44]. Wells argues that maternal pre-pregnancy nutritional status may be shaped by her own early growth in her natal household, which contributes to her ‘maternal capital,’ defined as phenotypic resources enabling investment in the offspring [45]. Thus child stunting may be more strongly influenced by an inter-generational process, which conceals any potential weaker association between decision-making and nutritional status. Furthermore, although stunting may not be directly associated with women’s decision-making power, it may be a proxy of broader inequitable gender norms [11, 46].

Misclassification of exposure may also be contributing to the lack of association between decision-making and child undernutrition observed in this study. The post-hoc analysis, for example, found that women who made decisions regarding her husband’s income jointly with her husband / partner or someone else had higher odds of having a stunted child than women who made such decisions alone. In addition, women who did not participate in decisions regarding her husband’s income had higher odds of having a stunted child than women who made such decisions alone. Though there may be advantages to sole decision-making regarding child undernutrition, excluding women who make decisions jointly with a husband / partner or someone else from the exposure variable may exclude women who contribute significantly to the decision-making process in a similar manner as women who make decisions alone. This finding provides grounds for future research delving into the nuances of decision-making classifications.

The current study suggests that women’s decision-making power, as captured by the 2013–2014 DHS survey, is not associated with child undernutrition in the DRC. Further research investigating decision-making power and child undernutrition is warranted as more detailed and context-specific measurement of decision-making may be required to better understand whether an association between women’s decision-making and child nutritional status exists. Moreover, it would be interesting to further explore decision-making as a potential predictor of stunting and wasting in children according to province and place of residence. Finally, the development of a consistent definition of decision-making will be important in allowing for cross-study comparisons to be made.

### Limitations and strengths

Results must be interpreted within the context of the study’s limitations. First, this study relied on DHS data, using self-reported information that is subject to recall and social desirability bias. Second, as an exposure variable decision-making is subject to misclassification bias since nuances regarding the degree of women’s participation in decision-making were not captured in the current study and it cannot be assumed that joint decision-making means equal decision-making power for men and women. A study investigating decision-making regarding women’s healthcare in Asia noted that joint decision-making should be interpreted cautiously in societies with low gender equality since women may be pressured / forced to agree with their husbands or partners [47]. Despite these challenges, some researchers argue that measuring joint decision-making is valuable as it may be associated with better health and socioeconomic outcomes than decision-making alone [48]. In addition to providing some clarity around this issue, future research should also investigate the association between husband’s decision-making and child undernutrition to understand potential gendered impact of decision-making. Third, given the cross-sectional nature, this study can only examine associations, not temporality. Fourth, covariates including the mother’s BMI, the child’s birth order, how long the child was breastfed, the mother’s nutritional status during pregnancy, gender equality in society, and parental occupations were not investigated as potential confounders, although they may be potential risk factors for undernutrition [10, 11, 35, 43]. Finally, this study did not investigate underweight (low weight-for-age) as an indicator of undernutrition. Given that the relationship between decision-making power and undernutrition is unclear in the DRC, we chose measures that were more indicative of chronic (stunting) and acute (wasting) undernutrition to better delineate potential causal relationships.

This study has several important strengths, including its large sample size, the fact that it is representative and therefore generalizable in the DRC, and its exceptionally high response rate. Furthermore, given the detailed survey, the analysis was also able to evaluate and control for a wide range of known and potential confounding variables.

This study contributes to the body of knowledge regarding women’s decision-making power in relation to undernutrition in the DRC. In this study, we did not observe any association between five indicators of women’s decision-making power and child undernutrition as currently measured in the DHS questionnaires. Future iterations of the DHS surveys may consider decisions regarding women’s mobility, daily household purchases, and child healthcare, as well as delving into the nuances of joint decision-making.

## Supporting information

S1 Table 2013–14 DRC-DHS questions. Questions used to collect data on decision-making, anthropometry, and covariates.
(DOCX)

S2 Table Stratified analysis by eastern and western provinces of the DRC. Logistic regression stratified by western Congolese provinces of Kinshasa, Bandundu, Bas-Congo, Equateur, Kasai-Oriental, and Kasai-Occidental, and by eastern Congolese provinces of Katanga, Maniema, North-Kivu, Orientale, and South-Kivu.
(DOCX)

S3 Table Post-hoc analysis of the association between joint decision making compared with women’s decision making alone on stunting and wasting. (DOCX)

S4 Table Post-hoc analysis of the association between no participation in decision-making compared to women’s decision making alone on stunting and wasting. (DOCX)

## Abbreviations

DRC: Democratic Republic of the Congo
DHS: Demographic and Health Survey
HAZ: height-for-age Z-score
SD: standard deviation
SPSS: Statistical Package for the Social Sciences
WHO: World Health Organization
WHZ: weight-for-height Z-score
DRC: Democratic Republic of the Congo
