# Supplementary material for: Women’s decision-making power and undernutrition in their children under age five in the Democratic Republic of the Congo: A cross-sectional study
Source: PLoS One. 2019 Dec 6;14(12):e0226041. doi: 10.1371/journal.pone.0226041 (PMC6897415; doi:10.1371/journal.pone.0226041)
Supplement: S1 Table — Questions used to collect data on decision-making, anthropometry, and covariates. (DOCX) [file pone.0226041.s001.docx]

**S1 Table.** **2013-14 DRC-DHS questions**. Questions used to collect data on decision-making, anthropometry, and covariates.

| **Variable** | **DHS Survey Question^b^** |
| --- | --- |
| **Decision-making^a^** | |
| Own income | (W919) Who usually decides how the money you earn will be used? |
| Husband/partner’s income | (W921) Who usually decides how your (husband’s/partner’s) money will be used? |
| Own healthcare | (W922) Who usually makes decisions about health care for yourself? |
| Household purchases | (W923) Who usually makes decisions about making major household purchases? |
| Visits to family | (W924) Who usually makes decisions about visits to your family or relatives? |
| **Anthropometry measures** | |
| Age | (B103) age in years |
| Weight | (B105) weight in kg |
| Height | (B106) height in cm |
| **Covariates** | |
| Child’s sex | (B8) current age of child in singleton years |
| Child’s age | (B4) sex of child |
| Mother’s education | (W108) What is the highest level you attended: none, primary, secondary, or higher? |
| Mother’s age | (V012) Current age in completed years is calculated from the century month code of the date of birth of the respondent (V011) and the century month code of the date of interview (V008). |
| Preceding birth interval | (B11) Preceding birth interval is calculated as the difference in months between the current birth and the previous birth, counting twins as one birth. In the DHS VII recode, B11 is also based on the CDC of date of birth of the children (B18). In previous recodes B11 was based on the CMC date of birth of the children (B3). |
| Number of children under five in household | (HVUnder5) Number of children under five was determined by summing the number of household members age 0-4 (HV105). |
| Number of people in household | (V136) Total number of household members is the number of usual residents (HV012) plus the number of visitors who slept in the house the previous night (HV013) that were listed in the household schedule. |
| Province | (HV024) Region of residence in which the household resides. Codes are country-specific. |
| Place of residence | (HV025) Type of place of residence where the household resides as either urban or rural. |
| Household socioeconomic status  (Wealth Index) | (V190) The wealth index is a composite measure of a household's cumulative living standard. The wealth index is calculated using easy-to-collect data on a household’s ownership of selected assets, such as televisions and bicycles; materials used for housing construction; and types of water access and sanitation facilities. |

^a^ Response codes

1 = Respondent alone

2 = Respondent and husband/partner

3 = Respondent and other person

4 = Husband/partner alone

5 = Someone else

6 = Other

^b^ W=Women’s Questionnaire, B=Biomarker Survey, V/HV=DHS Recode
