# Supplementary material for: Women’s decision-making power and undernutrition in their children under age five in the Democratic Republic of the Congo: A cross-sectional study
Source: PLoS One. 2019 Dec 6;14(12):e0226041. doi: 10.1371/journal.pone.0226041 (PMC6897415; doi:10.1371/journal.pone.0226041)
Supplement: S2 Table — Logistic regression stratified by western Congolese provinces of Kinshasa, Bandundu, Bas-Congo, Equateur, Kasai-Oriental, and Kasai-Occidental, and by eastern Congolese provinces of Katanga, Maniema, North-Kivu, Orientale, and South-Kivu. (DOCX) [file pone.0226041.s002.docx]

**S2A Table.** **Stratified analysis by eastern and western provinces of the DRC.** Logistic regression stratified by eastern Congolese provinces of Katanga, Maniema, North-Kivu, Orientale, and South-Kivu.

| **Eastern Provinces and the Outcome of Stunting** | | | | | |
| --- | --- | --- | --- | --- | --- |
| Covariate | Regarding her own income^a^ | Regarding her husband’s income^b^ | Regarding her own health care^b^ | Regarding major household purchases^b^ | Regarding visits to family^b^ |
| Participates in decision making  Yes  No | 1.0  0.73 (0.37, 1.43) | 1.0  0.97 (0.70, 1.33) | 1.0  1.16 (0.86, 1.56) | 1.0  1.10 (0.80, 1.52) | 1.0  1.11 (0.82, 1.51) |
| Child’s sex  Male  Female | 1.0  0.73 (0.49, 1.08) | 1.0  0.78 (0.58, 1.05) | 1.0  0.78 (0.58, 1.05) | 1.0  0.78 (0.58, 1.05) | 1.0  0.77 (0.57, 1.04) |
| Child’s age in years  0  1  2  3  4 | 1.0  2.38 (1.45, 3.91)*  2.92 (1.69, 5.06)*  3.84 (1.59, 9.25)*  8.33 (2.92, 23.76)* | 1.0  2.65 (1.74, 4.04)*  3.53 (2.13, 5.85)*  5.36 (2.71, 10.58)*  4.60 (2.31, 9.14)* | 1.0  2.75 (1.80 ,4.19)*  3.67 (2.22, 6.07)*  5.64 (2.87, 11.10)*  4.76 (2.37, 9.58)* | 1.0  2.75 (1.79, 4.23)*  3.66 (2.20, 6.09)*  5.59 (2.85, 10.94)*  4.68 (2.35, 9.31)* | 1.0  2.72 (1.78, 4.15)*  3.65 (2.19, 6.06)*  5.63 (2.86, 11.08)*  4.66 (2.38, 9.12)* |
| Mother’s age in years  15-19  20-24  25-29  30-34  35-39  40-44  45-49 | 1.0  0.48 (0.22, 1.06)  0.41 (0.21, 0.80)*  0.38 (0.16, 0.91)*  0.35 (0.13, 0.94)*  0.38 (0.14, 1.04)  0.10 (0.02, 0.42)* |  |  |  |  |
| Number of children under 5 in household  ≤1  2  ≥3 | 1.0  1.26 (0.58, 2.75)  2.09 (1.00, 4.35)* |  |  |  |  |
| Province  Katanga  Maniema  North-Kivu  Orientale  South-Kivu | 1.0  0.31 (0.13, 0.75)*  0.65 (034, 1.23)  0.61 (0.34, 1.08)  1.29 (0.70, 2.36) | 1.0  0.50 (0.28, 0.88)*  0.79 (0.48, 1.30)  0.61 (0.39, 0.96)*  1.23 (0.73, 2.08) | 1.0  0.49 (0.28, 0.86)*  0.83 (0.50, 1.37)  0.62 (0.39, 0.98)*  1.29 (0.75, 2.20) | 1.0  0.49 (0.28, 0.87)*  0.81 (0.49, 1.32)  0.62 (0.39, 0.98)*  1.30 (0.77, 2.19) | 1.0  0.49 (0.28, 0.87)*  0.82 (0.50, 1.34)  0.61 (0.39, 0.96)*  1.27 (0.75, 2.16) |
| Household economic status  Poorest  Poorer  Middle  Richer  Richest | 1.0  1.11 (0.59, 2.07)  0.98 (0.55, 1.76)  0.81 (0.39, 1.69)  0.37 (0.18, 0.78)* | 1.0  1.28 (0.78, 2.11)  0.97 (0.60, 1.58)  0.91 (0.53, 1.56)  0.37 (0.20, 0.71)* | 1.0  1.27 (0.78, 2.09)  0.96 (0.59, 1.56)  0.91 (0.53, 1.56)  0.36 (0.19, 0.69)* | 1.0  1.26 (0.77, 2.06)  0.97 (0.60, 1.56)  0.90 (0.52, 1.54)  0.37 (0.19, 0.70)* | 1.0  1.26 (0.77, 2.07)  0.95 (0.59, 1.55)  0.90 (0.52, 1.55)  0.36 (0.19, 0.69)* |
| **Eastern Provinces and the Outcome of Wasting** | | | | | |
|  |  |  |  |  |  |
| Covariate | Regarding her own income^c^ | Regarding her husband’s income^c^ | Regarding her own health care^c^ | Regarding major household purchases^d^ | Regarding visits to family^d^ |
| Participates in decision making  Yes  No | 1.0  0.79 (0.39, 1.59) | 1.0  0.90 (0.50, 1.62) | 1.0  0.92 (0.49, 1.72) | 1.0  0.90 (0.49, 1.66) | 1.0  0.71 (0.40, 1.27) |
| Child’s sex  Male  Female | 1.0  0.61 (0.26, 1.43) | 1.0  0.70 (0.37, 1.33) | 1.0  0.71 (0.38, 1.32) | 1.0  0.72 (0.36, 1.42) | 1.0  0.70 (0.36, 1.38) |
| Child’s age in years  0  1  2  3  4 | 1.0  0.48 (0.23, 1.00)*  0.61 (0.28, 1.32)  0.18 (0.03, 1.00*  0.09 (0.01, 0.72)* | 1.0  0.64 (0.34, 1.19)  0.87 (0.41, 1.85)  0.47 (0.15, 1.48)  0.08 (0.01, 0.64)* | 1.0  0.64 (0.34, 1.22)  0.87 (0.40, 1.87)  0.47 (0.15, 1.49)  0.08 (0.01, 0.65)* | 1.0  0.65 (0.35, 1.22)  0.87 (0.41, 1.83)  0.52 (0.17, 1.57)  0.09 (0.01, 0.72)* | 1.0  0.65 (0.35, 1.20)  0.87 (0.42, 1.80)  0.50 (0.16, 1.51)  0.09 (0.01, 0.74)* |
| Mother’s education  None  Primary  Secondary  Higher |  |  |  | 1.0  1.97 (1.13, 3.43)*  0.87 (0.45, 1.70)  6.03 (0.64, 57.36) | 1.0  1.96 (1.13, 3.38)*  0.89 (0.45, 1.74)  5.45 (0.57, 52.00) |
| Type of place of residence  Urban  Rural | 1.0  3.63 (1.14, 11.55)* | 1.0  2.43 (1.02, 5.81)* | 1.0  2.39 (1.02, 5.62)* | 1.0  2.27 (0.94, 5.52) | 1.0  2.30 (0.94, 5.61) |
| Province  Katanga  Maniema  North-Kivu  Orientale  South-Kivu | 1.0  4.02 (1.69, 9.57)*  0.42 (0.12, 1.47)  0.58 (0.25, 1.34)  0.50 (0.25, 1.03) | 1.0  2.26 (1.13, 4.54)*  0.59 (0.21, 1.66)  0.68 (0.34, 1.34)  0.54 (0.28, 1.02) | 1.0  2.26 (1.11, 4.60)*  0.59 (0.20, 1.71)  0.68 (0.34, 1.35)  0.54 (0.29, 1.01) | 1.0  2.38 (1.22, 4.63)*  0.62 (0.22, 1.78)  0.68 (0.34, 1.36)  0.56 (0.29, 1.07) | 1.0  2.37 (1.18, 4.74)*  0.59 (0.20, 1.73)  0.67 (0.34, 1.30)  0.52 (0.27, 0.97)* |
| Household economic status  Poorest  Poorer  Middle  Richer  Richest | 1.0  0.98 (0.44, 2.16)  1.39 (0.75, 2.58)  1.43 (0.63, 3.27)  1.29 (0.23, 7.10) | 1.0  0.63 (0.34, 1.19)  1.06 (0.59, 1.89)  1.00 (0.49, 2.04)  0.54 (0.15, 1.97) | 1.0  0.66 (0.35, 1.24)  1.10 (0.52, 2.12)  1.05 (0.52, 2.12)  0.56 (0.15, 2.04) | 1.0  0.61 (0.33, 1.15)  1.02 (0.57, 1.80)  1.03 (0.54, 1.98)  0.59 (0.16, 2.24) | 1.0  0.61 (0.32, 1.14)  1.01 (0.57, 1.77)  1.02 (0.53, 1.95)  0.60 (0.16, 2.26) |

* Represents a statistically significant finding

^a^ controlling for child’s sex, child’s age, household socioeconomic status, province, mother’s age, number of children under five in household

^b^ controlling for child’s sex, child’s age, household socioeconomic status, province

^c^ controlling for child’s sex, child’s age, household socioeconomic status, province, type of place of residence

^d^ controlling for child’s sex, child’s age, household socioeconomic status, province, type of place of residence, mother’s education

**S2B Table.** **Stratified analysis by eastern and western provinces of the DRC**. Logistic regression stratified by eastern Congolese provinces of Kinshasa, Bandundu, Bas-Congo, Equateur, Kasai-Oriental, and Kasai-Occidental.

| **Western Provinces and the Outcome of Stunting** | | | | | |
| --- | --- | --- | --- | --- | --- |
| Covariate | Regarding her own income^a^ | Regarding her husband’s income^b^ | Regarding her own health care^b^ | Regarding major household purchases^b^ | Regarding visits to family^b^ |
| Participates in decision making  Yes  No | 1.0  0.98 (0.73, 1.33) | 1.0  1.28 (1.00, 1.63)* | 1.0  1.06 (0.79, 1.41) | 1.0  1.05 (0.80, 1.37) | 1.0  0.93 (0.70, 1.22) |
| Child’s sex  Male  Female | 1.0  0.65 (0.48, 0.89)* | 1.0  0.78 (0.60, 1.01) | 1.0  0.78 (0.61, 1.01) | 1.0  0.78 (0.60, 1.00)* | 1.0  0.78 (0.60, 1.00) |
| Child’s age in years  0  1  2  3  4 | 1.0  3.68 (0.25, 5.36)*  9.11 (5.67, 14.64)*  12.45 (6.41, 24.18)*  9.67 (4.41, 21.24)* | 1.0  4.42 (3.09, 6.32)*  8.76 (5.66, 13.56)*  8.60 (5.22, 14.18)*  7.00 (3.55, 13.82)* | 1.0  4.19 (2.91, 6.05)*  8.15 (5.17, 12.86)*  8.37 (5.09, 13.78)*  6.55 (3.37, 12.72)* | 1.0  4.20 (2.91, 6.06)  8.28 (5.25, 13.07)*  8.41 (5.10, 13.87)*  6.55 (3.35, 12.79)* | 1.0  4.18 (2.89, 6.04)*  8.10 (5.13, 12.79)*  8.30 (5.03, 13.69)*  6.52 (3.33, 12.75)* |
| Mother’s age in years  15-19  20-24  25-29  30-34  35-39  40-44  45-49 | 1.0  1.56 (0.73, 3.35)  0.99 (0.46, 2.15)  1.30 (0.64, 2.66)  1.20 (0.54, 2.70)  0.93 (0.40, 2.12)  0.40 (0.11, 1.40) |  |  |  |  |
| Number of children under 5 in household  ≤1  2  ≥3 | 1.0  1.45 (1.07, 1.97)*  1.65 (1.01, 2.68)* |  |  |  |  |
| Province  Kinshasa  Bandundu  Bas-Congo  Equateur  Kasi-Occidental  Kasi-Oriental | 1.0  0.93 (0.39, 2.24)  2.14 (0.86, 5.30)  0.94 (0.39, 2.27)  0.48 (0.58, 3.77)  1.68 (0.72, 3.91) | 1.0  0.86 (0.38, 1.93)  1.92 (0.84, 4.39)  0.75 (0.33, 1.70)  1.73 (0.71, 4.22)  1.61 (0.75, 3.47) | 1.0  0.93 (0.42, 2.08)  2.06 (0.89, 4.75)  0.82 (0.36, 1.85)  1.84 (0.76, 4.46)  1.78 (0.83, 3.79) | 1.0  0.91 (0.41, 2.02)  2.15 (0.95, 4.87)  0.81 (0.36, 1.80)  1.79 (0.74, 4.33)  1.74 (0.82, 3.71) | 1.0  0.94 (0.42, 2.08)  2.09 (0.91, 4.81)  0.83 (0.37, 1.86)  1.87 (0.77, 4.53)  1.82 (0.85, 3.86) |
| Type of place of residence  Urban  Rural |  | 1.0  1.36 (0.98, 1.88) | 1.0  1.40 (1.02, 1.92)* | 1.0  1.39 (1.01, 1.91)* | 1.0  1.41 (1.02, 1.94)* |
| Household economic status  Poorest  Poorer  Middle  Richer  Richest | 1.0  0.81 (0.52, 1.25)  0.70 (0.47, 1.06)  0.41 (0.23, 0.73)*  0.18 (0.08, 0.38)* | 1.0  0.73 (0.50, 1.05)  0.78 (0.56, 1.08)  0.46 (0.28, 0.76)*  0.28 (0.14, 0.60)* | 1.0  0.74 (0.51, 1.07)  0.77 (0.56, 1.06)  0.46 (0.28, 0.77)*  0.30 (0.14, 0.62)* | 1.0  0.74 (0.51, 1.08)  0.78 (0.57, 1.08)  0.46 (0.28, 0.77)*  0.29 (0.14, 0.60* | 1.0  0.74 (0.50, 1.07)  0.77 (0.56, 1.06)  0.46 (0.28, 0.77)*  0.29 (0.14, 0.61)* |
| **Western Provinces and the Outcome of Wasting** | | | | | |
|  |  |  |  |  |  |
| Covariate | Regarding her own income^c^ | Regarding her husband’s income^d^ | Regarding her own health care^d^ | Regarding major household purchases^d^ | Regarding visits to family^d^ |
| Participates in decision making  Yes  No | 1.0  1.30 (0.66, 2.58) | 1.0  1.23 (0.75, 2.03) | 1.0  1.00 (0.62, 1.62) | 1.0  0.85 (0.58, 1.24) | 1.0  0.92 (0.60, 1.40) |
| Child’s sex  Male  Female | 1.0  1.10 (0.70, 1.72) | 1.0  0.83 (0.57, 1.21) | 1.0  0.82 (0.56, 1.20) | 1.0  0.82 (0.56, 1.20) | 1.0  0.82 (0.56, 1.20) |
| Child’s age in years  0  1  2  3  4 | 1.0  0.56 (0.26, 1.19)  0.67 (0.30, 1.51)  0.64 (0.23, 1.79)  0.41 (0.12, 1.44) | 1.0  0.55 (0.31, 0.99)*  0.59 (0.32, 1.10)  0.56 (0.24, 1.31)  0.34 (0.10, 1.19) | 1.0  0.57 (0.32, 1.01)  0.58 (0.31, 1.09)  0.56 (0.24, 1.29)  0.34 (0.10, 1.18) | 1.0  0.57 (0.32, 1.01)  0.58 (0.31, 1.09)  0.54 (0.23, 1.28)  0.34 (0.10, 1.18) | 1.0  0.57 (0.32, 1.01)  0.58 (0.31, 1.09)  0.55 (0.23, 1.29)  0.34 (0.10, 1.17) |
| Preceding birth interval  0-23 months  > 24 months |  | 1.0  0.66 (0.42, 1.04) | 1.0  0.67 (0.43, 1.04) | 1.0  0.67 (0.43, 1.05) | 1.0  0.67 (0.42, 1.05) |
| Province  Kinshasa  Bandundu  Bas-Congo  Equateur  Kasi-Occidental  Kasi-Oriental | 1.0  0.68 (0.18, 2.64)  0.68 (0.17, 2.69)  0.27 (0.07, 1.04)  0.43 (0.11, 1.71)  0.32 (0.09, 1.20) |  |  |  |  |
| Household economic status  Poorest  Poorer  Middle  Richer  Richest | 1.0  1.43 (0.80, 2.55)  1.26 (0.58, 2.75)  1.45 (0.65, 3.23)  0.21 (0.07, 0.65)* | 1.0  1.03 (0.53, 2.01)  0.90 (0.47, 1.72)  1.24 (0.63, 2.46)  0.55 (0.25, 1.20) | 1.0  1.03 (0.53, 2.01)  0.92 (0.49, 1.73)  1.23 (0.62, 2.44)  0.52 (0.24, 1.13) | 1.0  1.03 (0.53, 2.00)  0.91 (0.48, 1.72)  1.21 (0.62, 2.39)  0.51 (0.24, 1.09) | 1.0  1.03 (0.53, 1.99)  0.92 (0.49, 1.73)  1.22 (0.62, 2.42)  0.52 (0.24, 1.11) |

* Represents a statistically significant finding

^a^ controlling for child’s sex, child’s age, household socioeconomic status, province, mother’s age, number of children under five in household

^b^ controlling for child’s sex, child’s age, household socioeconomic status, province, type of place of residence

^c^ controlling for child’s sex, child’s age, household socioeconomic status, province

^d^ controlling for child’s sex, child’s age, household socioeconomic status, preceding birth interval
