# Supplementary material for: Women’s decision-making power and undernutrition in their children under age five in the Democratic Republic of the Congo: A cross-sectional study
Source: PLoS One. 2019 Dec 6;14(12):e0226041. doi: 10.1371/journal.pone.0226041 (PMC6897415; doi:10.1371/journal.pone.0226041)
Supplement: S3 Table — (DOCX) [file pone.0226041.s003.docx]

**S3A Table. Post-hoc analysis of the association between joint decision making compared with women’s decision making alone on stunting.**

| **Covariate** | **Regarding her own income^a^** | **Regarding her husband’s income^b^** | **Regarding her own health care^a^** | **Regarding major household purchases^a^** | **Regarding visits to family^c^** |
| --- | --- | --- | --- | --- | --- |
| Participates in decision making  Alone  Joint | 1.0  1.10 (0.81, 1.50) | 1.0  1.59 (1.06, 2.40)* | 1.0  1.31 (0.84, 2.06) | 1.0  1.10 (0.79, 1.54) | 1.0  0.96 (0.68, 1.36) |
| Child’s sex  Male  Female | 1.0  0.75 (0.56, 0.99)* | 1.0  0.84 (0.63, 1.12) | 1.0  0.76 (0.57, 1.03) | 1.0  0.84 (0.65, 1.08) | 1.0  0.71 (0.52, 0.96)* |
| Child’s age in years  0  1  2  3  4 | 1.0  2.56 (1.82, 3.60)*  4.19 (2.74, 6.43)*  4.13 (2.47, 6.90)*  3.83 (1.95, 7.52)* | 1.0  3.03 (2.19, 4.21)*  4.71 (3.22, 6.88)*  8.30 (4.97, 13.87)*  5.53 (2.87, 10.68)* | 1.0  3.25 (2.22, 4.76)*  4.72 (3.01, 7.42)*  5.02 (2.57, 9.80)*  4.15 (1.83, 9.43)* | 1.0  2.90 (2.01, 4.18)*  5.03 (3.26, 7.77)*  6.45 (4.11, 10.12)*  4.41 (2.34, 8.32)* | 1.0  3.14 (2.13, 4.62)*  6.49 (3.91, 10.77)*  6.79 (3.61, 12.77)*  3.50 (1.80, 6.79)* |
| Mother’s education  None  Primary  Secondary  Higher |  | 1.0  1.30 (0.92, 1.83)  0.83 (0.54, 1.27)  0.53 (0.14, 1.97) |  |  | 1.0  1.39 (0.95, 2.04)  0.73 (0.45, 1.19)  0.61 (0.11, 3.24) |
| Preceding Birth Interval  0-23 mo  > 24 mo |  |  |  |  | 1.0  0.71 (0.48, 1.05) |
| Province  Kinshasa  Bandundu  Bas-Congo  Equateur  Kasi-Occidental  Kasi-Oriental  Katanga  Maniema  North-Kivu  Orientale  South-Kivu | 1.0  1.31 (0.58, 2.94)  2.41 (1.04, 5.60)*  1.35 (0.62, 2.94)  2.04 (0.85, 4.91)  2.22 (1.06, 4.65)*  2.20 (1.02, 4.73)*  0.67 (0.20, 2.33)  1.62 (0.58, 4.50)  1.73 (0.76, 3.94)  4.91 (1.74, 13.85)* | 1.0  1.14 (0.54, 2.40)  1.77 (0.78, 4.02)  0.96 (0.48, 1.94)  1.63 (0.76, 3.50)  1.56 (0.78, 3.12)  2.30 (1.12, 4.72)*  0.92 (0.33, 2.54)  1.42 (0.61, 3.30)  1.29 (0.60, 2.76)  3.50 (1.70, 7.18)* | 1.0  0.88 (0.36, 2.18)  1.82 (0.68, 4.84)  0.89 (0.36, 2.24)  1.05 (0.39, 2.82)  1.60 (0.70, 3.65)  1.67 (0.71, 3.94)  0.85 (0.27, 2.68)  1.35 (0.57, 3.17)  0.96 (0.40, 2.31)  2.25 (0.89, 5.66) | 1.0  1.50 (0.69, 3.29)  2.99 (1.12, 7.96)  1.35 (0.62, 2.92)  1.67 (0.69, 4.02)  2.19 (1.07, 4.49)*  2.50 (1.16, 5.41)*  0.97 (0.36, 2.61)  1.81 (0.74, 4.44)  1.51 (0.66, 3.47)  4.49 (1.95, 10.31)* | 1.0  0.83 (0.31, 2.21)  1.73 (0.66, 4.57)  0.85 (0.33, 2.22)  0.91 (0.28, 2.91)  2.19 (0.82, 5.87)  1.69 (0.63, 4.55)  1.09 (0.33, 3.63)  0.97 (0.38, 2.46)  0.91 (0.30, 2.74)  2.24 (0.85, 5.87) |
| Household economic status  Poorest  Poorer  Middle  Richer  Richest | 1.0  0.89 (0.56, 1.40)  0.96 (0.61, 1.51)  0.67 (0.37, 1.20)  0.35 (0.19, 0.64)* | 1.0  0.98 (0.65, 1.47)  0.89 (0.58, 1.37)  0.69 (0.42, 1.13)  0.41 (0.23, 0.72)* | 1.0  1.03 (0.69, 1.55)  0.76 (0.51, 1.13)  0.53 (0.34, 0.85)*  0.26 (0.14, 0.49)* | 1.0  1.07 (0.77, 1.48)  0.84 (0.58, 1.21)  0.66 (0.41, 1.05)  0.37 (0.22, 0.64)* | 1.0  1.11 (0.73, 1.66)  1.03 (0.68, 1.56)  0.76 (0.46, 1.26)  0.31 (0.15, 0.65)* |

* Represents a statistically significant finding

^a^ controlling for child’s sex, child’s age, household socioeconomic status, province

^b^ controlling for child’s sex, child’s age, household socioeconomic status, province, mother’s education

^c^ controlling for child’s sex, child’s age, household socioeconomic status, province, mother’s education, preceding birth interval

**S3B Table. Post-hoc analysis of the association between joint decision making compared with women’s decision making alone on wasting.**

|  | **COR: 1.04 (0.62, 1.73)** | **COR: 0.94 (0.53, 1.66)** | **COR: 0.85 (0.51, 1.45)** | **COR: 0.94 (0.54, 1.68)** | **COR: 0.90 (0.60, 1.35)** |
| --- | --- | --- | --- | --- | --- |
| **Covariate** | **Regarding her own income^a^** | **Regarding her husband’s income^a^** | **Regarding her own health care^a^** | **Regarding major household purchases^b^** | **Regarding visits to family^c^** |
| Participates in decision making  Alone  Joint | 1.0  0.86 (0.51, 1.47) | 1.0  0.81 (0.46, 1.42) | 1.0  0.68 (0.41, 1.14) | 1.0  1.02 (0.60, 1.72) | 1.0  0.86 (0.57, 1.31) |
| Child’s sex  Male  Female | 1.0  0.79 (0.46, 1.34) | 1.0  0.86 (0.53, 1.41) | 1.0  0.70 (0.41, 1.20) | 1.0  0.99 (0.63, 1.55) | 1.0  0.78 (0.49, 1.24) |
| Child’s age in years  0  1  2  3  4 | 1.0  0.71 (0.41, 1.22)  1.01 (0.54, 1.89)  0.65 (0.25, 1.73)  0.49 (0.15, 1.57) | 1.0  0.54 (0.32, 0.93)*  0.81 (0.47, 1.41)  0.41 (0.18, 0.94)*  0.16 (0.05, 0.58)* | 1.0  0.40 (0.23, 0.70)*  0.67 (0.37, 1.24)  0.60 (0.26, 1.38)  0.13 (0.03, 0.64)* | 1.0  0.57 (0.35, 0.94)*  0.66 (0.37, 1.18)  0.49 (0.22, 1.10)  0.22 (0.07, 0.69)* | 1.0  0.42 (0.24, 0.74)*  0.65 (0.33, 1.29)  0.51 (0.23, 1.16)  0.37 (0.10, 1.34) |
| Mother’s education  None  Primary  Secondary  Higher |  |  |  |  | 1.0  1.87 (1.11, 3.15)*  1.34 (0.68, 2.66)  1.54 (0.24, 10.00) |
| Province  Kinshasa  Bandundu  Bas-Congo  Equateur  Kasi-Occidental  Kasi-Oriental  Katanga  Maniema  North-Kivu  Orientale  South-Kivu |  |  |  | 1.0  0.62 (0.19, 2.02)  0.80 (0.21, 3.00)  0.39 (0.12, 1.26)  0.32 (0.08, 1.29)  0.54 (0.18, 1.65)  0.39 (0.12, 1.24)  1.15 (0.26, 5.04)  0.16 (0.04, 0.59)*  0.46 (0.13, 1.60)  0.36 (0.11, 1.17) |  |
| Household economic status  Poorest  Poorer  Middle  Richer  Richest | 1.0  1.19 (0.65, 2.20)  1.76 (0.94, 3.32)  1.09 (0.52, 2.30)  0.42 (0.14, 1.22) | 1.0  1.04 (0.61, 1.80)  0.89 (0.48, 1.67)  0.86 (0.45, 1.62)  0.35 (0.17, 0.74)* | 1.0  1.14 (0.66, 1.96)  1.03 (0.52, 2.03)  0.98 (0.48, 2.00)  0.41 (0.17, 0.97)* | 1.0  1.48 (0.91, 2.41)  1.27 (0.70, 2.30)  1.03 (0.54, 1.97)  0.32 (0.13, 0.78)* | 1.0  1.25 (0.67, 2.33)  1.14 (0.59, 2.23)  0.80 (0.42, 1.55)  0.33 (0.12, 0.86)* |

* Represents a statistically significant finding

^a^ controlling for child’s sex, child’s age, household socioeconomic status

^b^ controlling for child’s sex, child’s age, household socioeconomic status, province

^c^ controlling for child’s sex, child’s age, household socioeconomic status mother’s education,
