# Supplementary material for: Women’s decision-making power and undernutrition in their children under age five in the Democratic Republic of the Congo: A cross-sectional study
Source: PLoS One. 2019 Dec 6;14(12):e0226041. doi: 10.1371/journal.pone.0226041 (PMC6897415; doi:10.1371/journal.pone.0226041)
Supplement: S4 Table — (DOCX) [file pone.0226041.s004.docx]

**S4A Table. Post-hoc analysis of the association between no participation in decision-making compared to women’s decision making alone on stunting.**

| **Covariate** | **Regarding her own income^a^** | **Regarding her husband’s income^b^** | **Regarding her own health care^b^** | **Regarding major household purchases^b^** | **Regarding visits to family^c^** |
| --- | --- | --- | --- | --- | --- |
| Participates in decision making  Alone  No participation | 1.0  1.11 (0.78, 1.58) | 1.0  1.71 (1.14, 2.57)* | 1.0  1.39 (0.91, 2.11) | 1.0  0.97 (0.69, 1.35) | 1.0  1.08 (0.81, 1.44) |
| Child’s sex  Male  Female | 1.0  0.54 (0.38, 0.76)* | 1.0  0.78 (0.58, 1.05) | 1.0  0.82 (0.64, 1.06) | 1.0  0.77 (0.58, 1.02) | 1.0  0.87 (0.66, 1.14) |
| Child’s age in years  0  1  2  3  4 | 1.0  2.92 (2.01, 4.25)*  5.04 (3.04, 8.34)*  4.91 (2.51, 9.62)*  8.13 (3.75, 17.66)* | 1.0  4.62 (3.08, 6.94)*  9.82 (5.82, 16.59)*  8.30 (4.46, 15.47)*  7.30 (3.57, 14.93)* | 1.0  3.86 (2.60, 5.73)*  7.87 (4.79, 12.91)*  10.63 (6.09, 18.56)*  8.42 (4.38, 16.17)* | 1.0  4.08 (2.85, 5.84)*  7.12 (4.42, 11.46)*  9.20 (5.14, 16.46)*  6.94 (3.31, 14.57)* | 1.0  3.69 (2.62, 5.20)*  5.58 (3.61, 8.62)*  8.23 (5.25, 12.90)*  5.73 (3.11, 10.54)* |
| Mother’s age in years  15-19  20-24  25-29  30-34  35-39  40-44  45-49 | 1.0  0.97 (0.28, 3.34)  0.59 (0.18, 1.96)  0.82 (0.25, 2.72)  0.78 (0.22, 2.75)  0.86 (0.24, 3.02)  0.17 (0.04, 0.77)* | 1.0  1.17 (0.60, 2.29)  0.58 (0.31, 1.08)  0.77 (0.42, 1.39)  0.74 (0.37, 1.50)  0.80 (0.41, 1.56)  0.59 (0.25, 1.41) | 1.0  1.29 (0.72, 2.33)  0.80 (0.44, 1.43)  1.03 (0.59, 1.80)  1.02 (0.54, 1.96)  1.05 (0.53, 2.05)  0.63 (0.24, 1.64) | 1.0  1.48 (0.82, 2.66)  0.72 (0.41, 1.27)  0.94 (0.54, 1.63)  11.14 (0.61, 2.10)  0.96 (0.51, 1.81)  0.46 (0.18, 1.20) |  |
| Preceding birth interval  0-23 mo  > 24 mo | 1.0  0.54 (0.35, 0.82)* |  |  |  |  |
| Number of children under 5 in household  0-1  2  3+ |  | 1.0  1.34 (0.96, 1.86)  1.97 (1.29, 3.03)* | 1.0  1.30 (0.96, 1.77)  1.62 (1.13, 2.33)* | 1.0  1.29 (0.93, 1.80)  1.61 (1.08, 2.41)* |  |
| Province  Kinshasa  Bandundu  Bas-Congo  Equateur  Kasi-Occidental  Kasi-Oriental  Katanga  Maniema  North-Kivu  Orientale  South-Kivu | 1.0  0.74 (0.23, 2.39)  2.47 (0.77, 7.96)  0.86 (0.26, 2.86)  1.33 (0.39, 4.54)  1.66 (0.51, 5.39)  1.80 (0.51, 6.37)  0.71 (0.14, 3.73)  1.27 (0.36, 4.47)  1.11 (0.33, 3.80)  0.95 (0.16, 5.72) | 1.0  0.57 (0.20, 1.62)  1.82 (0.59, 5.61)  0.53 (0.19, 1.48)  1.92 (0.62, 5.92)  1.18 (0.43, 3.29)  1.21 (0.42, 3.45)  0.49 (0.13, 1.78)  1.38 (0.41, 4.50)  0.74 (0.25, 2.24)  1.34 (0.41, 4.24) | 1.0  1.04 (0.45, 2.43)  2.32 (0.82, 6.52)  0.94 (0.39, 2.23)  2.89 (1.14, 7.30)  1.91 (0.82, 4.46)  2.47 (1.07, 5.73)*  0.96 (0.36, 2.55)  1.81 (0.57, 5.71)  1.32 (0.53, 3.29)  4.32 (1.55, 12.01)* | 1.0  0.70 (0.27, 1.85)  2.27 (0.79, 6.58)  0.66 (0.25, 1.79)  2.04 (0.73, 568)  1.46 (0.55, 3.84)  1.70 (0.65, 4.44)  0.86 (0.31, 2.39)  1.83 (0.65, 5.11)  0.88 (0.32, 2.41)  1.92 (0.66, 5.62) | 1.0  0.92 (0.39, 2.17)  2.06 (0.76, 5.54)  0.68 (0.28, 1.63)  2.12 (0.82, 5.47)  1.48 (0.63, 3.46)  1.95 (0.84, 4.53)  0.91 (0.37, 2.20)  1.96 (0.71, 5.43)  1.15 (0.46, 2.86)  2.49 (0.92, 6.78) |
| Household economic status  Poorest  Poorer  Middle  Richer  Richest | 1.0  0.97 (0.60, 1.57)  0.81 (0.49, 1.33)  0.50 (0.28, 0.88)*  0.25 (0.10, 0.62)* | 1.0  0.97 (0.64, 1.45)  0.85 (0.58, 1.25)  0.48 (0.31, 0.74)*  0.23 (0.11, 0.46)* | 1.0  0.86 (0.59, 1.25)  0.86 (0.60, 1.22)  0.62 (0.40, 0.97)*  0.27 (0.16, 0.47)* | 1.0  0.83 (0.55, 1.26)  0.76 (0.52, 1.10)  0.48 (0.31, 0.74)*  0.18 (0.09, 0.33)* | 1.0  0.83 (0.58, 1.18)  0.85 (0.61, 1.19)  0.43 (0.28, 0.67)*  0.30 (0.18, 0.53)* |

* Represents a statistically significant finding

^a^ controlling for child’s age, child’s sex, household socioeconomic status, province, mother’s age, preceding birth interval

^b^ controlling for child’s age, child’s sex, household socioeconomic status, province, mother’s age, number of children under five in household

^c^ controlling for child’s age, child’s sex, household socioeconomic status, province

**S4B Table. Post-hoc analysis of the association between no participation in decision-making compared to women’s decision making alone on wasting.**

| **Covariate** | **Regarding her own income^a^** | **Regarding her husband’s income^b^** | **Regarding her own health care^b^** | **Regarding major household purchases^b^** | **Regarding visits to family^b^** |
| --- | --- | --- | --- | --- | --- |
| Participates in decision making  Alone  No participation | 1.0  0.82 (0.49, 1.39) | 1.0  1.04 (0.58, 1.88) | 1.0  0.75 (0.44, 1.29) | 1.0  0.86 (0.53, 1.40) | 1.0  0.81 (0.55, 1.19) |
| Child’s sex  Male  Female | 1.0  1.06 (0.68, 1.63) | 1.0  0.74 (0.49, 1.12) | 1.0  0.84 (0.59, 1.20) | 1.0  0.69 (0.45, 1.05) | 1.0  0.82 (0.57, 1.17) |
| Child’s age in years  0  1  2  3  4 | 1.0  0.35 (0.16, 0.78)*  0.52 (0.22, 1.20)  0.44 (0.16, 1.22)  0.30 (0.07, 1.23) | 1.0  0.56 (0.29, 1.06)  0.64 (0.31, 1.34)  0.86 (0.38, 1.94)  0.27 (0.06, 1.33) | 1.0  0.63 (0.36, 1.11)  0.66 0.35, 1.25)  0.65 (0.29, 1.46)  0.27 (0.07, 1.03) | 1.0  0.59 (0.31, 1.12)  0.61 (0.29, 1.29)  0.87 (0.38, 1.98)  0.22 (0.05, 1.11) | 1.0  0.63 (0.38, 1.05)  0.62 (0.35, 1.13)  0.61 (0.27, 1.38)  0.08 (0.02, 0.36)* |
| Province  Kinshasa  Bandundu  Bas-Congo  Equateur  Kasi-Occidental  Kasi-Oriental  Katanga  Maniema  North-Kivu  Orientale  South-Kivu | 1.0  1.39 (0.15, 13.16)  1.55 (0.17, 14.61)  0.28 (0.03, 2.63)  0.39 (0.04, 3.65)  0.49 (0.05, 4.64)  0.76 (0.09, 6.65)  1.72 (0.16, 19.11)  0.13 (0.01, 1.73)  0.19 (0.02, 2.09)  0.41 (0.03, 5.20) | 1.0  3.46 (0.56, 21.40)  3.34 (0.57, 19.59)  1.26 (0.19, 8.22)  1.67 (0.23, 12.32)  1.41 (0.21, 9.29)  1.70 (0.30, 9.65)  3.01 (0.49, 18.44)  2.06 (0.24, 17.70)  0.73 (0.11, 4.77)  0.73 (0.10, 5.40) | 1.0  2.22 (0.67, 7.40)  1.93 (0.54, 6.88)  0.92 (0.26, 3.18)  1.20 (0.31, 4.70)  0.99 (0.28, 3.52)  1.21 (0.38, 3.79)  2.32 (0.71, 7.57)  1.10 (0.23, 5.38)  0.60 (0.16, 2.30)  0.33 (0.06, 1.92) | 1.0  3.04 (0.65, 14.30)  1.92 (0.39, 9.56)  1.33 (0.27, 6.49)  1.91 (0.36, 10.15)  1.36 (0.27, 6.78)  1.98 (0.46, 8.48)  2.91 (0.62, 13.64)  1.80 (0.26, 12.71)  0.79 (0.15, 4.13)  0.37 (0.04, 3.41) | 1.0  1.64 (0.50, 5.34)  1.58 (0.46, 5.44)  0.74 (0.21, 2.56)  0.82 (0.21, 3.19)  0.98 (0.21, 3.19)  1.01 (0.33, 3.10)  1.74 (0.55, 5.55)  0.82 (0.15, 4.51)  0.40 (0.10, 1.51)  0.19 (0.02, 1.55) |
| Type of place of residence  Urban  Rural | 1.0  2.10 (0.89, 4.99) |  |  |  |  |
| Household economic status  Poorest  Poorer  Middle  Richer  Richest | 1.0  1.24 (0.70, 2.21)  1.11 (0.56, 2.19)  1.29 (0.53, 3.11)  0.13 (0.03, 0.68)* | 1.0  0.63 (0.34 1.16)  0.88 (0.51, 1.53)  0.75 (0.36, 1.56)  0.40 (0.13, 1.26) | 1.0  0.62 (0.35, 1.09)  0.84 (0.50, 1.43)  0.66 (0.34, 1.29)  0.36 (0.15, 0.87)* | 1.0  0.56 (0.28, 1.11)  0.95 (0.54, 1.66)  0.77 (0.38, 1.56)  0.47 (0.16, 1.35) | 1.0  0.64 (0.37, 1.11)  0.75 (0.45, 1.26)  0.72 (0.37, 1.40)  0.37 (0.15, 0.91)* |

* Represents a statistically significant finding

^a^ controlling for child’s age, child’s sex, household socioeconomic status, province, type of place of residence

^b^ controlling for child’s age, child’s sex, household socioeconomic status, province
